# The Asthma Consultative Process: A Collaborative Approach to Integrating Genomics Into Public Health Practice

**Published:** 2005-03-15

**Authors:** Karen L Edwards, Tabitha A Harrison, Wylie Burke

**Affiliations:** University of Washington; Department of Epidemiology, School of Public Health and Community Medicine, University of Washington, Seattle, Wash; Department of Medical History and Ethics, School of Medicine, University of Washington, Seattle, Wash

## Abstract

Genomics research findings on asthma are reported with increasing frequency. As these findings are incorporated into existing knowledge of disease etiology and pathogenesis, the implications for public health practice need to be considered. In 2003, the University of Washington Center for Genomics and Public Health initiated a project to synthesize information about asthma genomics, to examine its relevance to public health research and practice, and to communicate findings to a public health practice audience. This goal was achieved through review of the scientific literature, formation of a working group, and consultations with professionals and community organizations. This paper describes the methods used to conduct these professional and community consultations, referred to as the asthma consultative process, and discusses the lessons learned from this activity.

## Introduction

There is widespread enthusiasm that findings from the Human Genome Project will significantly shape the public health practice of the future (1). In recent years, genomic research has provided new insights into the etiology and pathogenesis of asthma, a major public health burden in the United States. However, while genomics research is leading to new hypotheses about asthma onset and progression, and may alter how asthma is understood at the molecular level, it is not yet clear if and how genomics can be used in asthma prevention, diagnosis, and management. Additionally, the volume of new literature poses a potential barrier for professionals who wish to stay abreast of current findings. To translate basic sciences research findings into relevant applications, it will be important to gather and synthesize the rapidly changing body of information about asthma genomics and to consider research findings in the context of public health principles. Thus, a multidisciplinary approach for summarizing, evaluating, and translating knowledge in asthma genomics for public health practice is needed. To meet this challenge, the University of Washington Center for Genomics and Public Health (UWCGPH) developed a process for summarizing and considering potential implications of asthma genomics within the broad context of public health practice. This paper describes the methods used to conduct professional and community consultations, referred to as the asthma consultative process, and discusses the lessons learned from this activity.

## Laying the Groundwork for the Asthma Consultative Process

In December 2002, the UWCGPH initiated a project with support from the Centers for Disease Control and Prevention's (CDC's) Office of Genomics and Disease Prevention (OGDP) and National Center for Environmental Health (NCEH) to gather and synthesize information about asthma genomics, to examine its relevance to public health research and practice, and to translate findings into language appropriate for an audience of public health practitioners. The UWCGPH identified a need to examine the knowledge base in asthma genomics and to involve public health and genomics experts in the project to accomplish these goals. The UWCGPH met these needs through a yearlong project that involved an extensive review of the literature, the formation of a working group, and the asthma consultative process.

The project began with a review of the published scientific literature on asthma genomics. The UWCGPH identified relevant literature through an initial electronic search of PubMed using the search terms "asthma genomics (gene/genetic/genetics)," restricting the search to studies in humans and articles written in English. This literature base was used to identify current findings relevant to the integration of genomics into public health research and practice related to asthma. Periodic reviews of the literature were conducted, and additional publications, including local, national, and federal documents on asthma, were identified throughout the project period. Following the initial literature search, the UWCGPH drafted a document summarizing current asthma research priorities and evidence of genetic contributors to asthma risk.

In addition to the literature review, the UWCGPH formed a working group of 12 professionals knowledgeable in asthma, genomics, and public health practice who contributed expert opinion, provided guidance on how to structure consultations, and helped formulate project findings. Members of the group were selected to ensure broad representation of public health interests, including research and practice at the state and national levels.

## Asthma Consultative Frameworks

**Figure 1:**
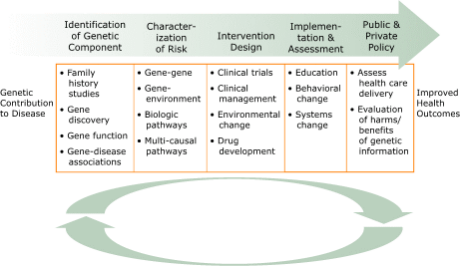
Translational pathway, which serves to illustrate translation of scientific findings into public health practice.

The Asthma Working Group developed two frameworks: 1) the translational pathway and 2) a matrix of perspectives and health interventions. Both frameworks were used to define stakeholders with an interest in genomics and to broadly characterize types of health care interventions in which genomics could be used. These frameworks also served as guides for focusing consultations to specific areas of expertise or practice. The translational pathway (Figure 1) served to illustrate translation of scientific findings into public health practice and to emphasize the importance of cross-disciplinary interactions in this process. Importantly, this pathway encouraged consultants to view their work as part of a broader effort to use genomics advances to improve health outcomes.

**Table:** A text description of this chart is also available

The broad spectrum of public health research and practice occurs in many settings, draws upon several disciplines, and affects the population's health through several means. Figure 2 presents a matrix of perspectives and health interventions, outlining four key opportunities for health intervention at different stages of disease prevention and management: population-based prevention, risk-based prevention, diagnosis, and management. In addition to designating areas of intervention, the framework outlines the perspectives of groups who have a vested interest in the research and use of genomics for health care purposes: individuals with asthma and their family members, communities, researchers, health care professionals, commercial developers, and public health practitioners. This framework was used to create discussion probes (Appendix) for the consultations and also helped guide the selection of experts who participated in the asthma consultative process.

**Figure 2:** A matrix of perspectives and health interventions, which outlines four key opportunities for health interventions at different stages of disease prevention and management.

## Asthma Consultative Process

The primary purpose of the asthma consultative process was to supplement scientific evidence identified in the medical literature and to provide comment on the potential implications genomics may have on public health efforts in asthma. The selection of consultants was guided by the goal of representing a broad range of perspectives. Therefore, we conducted a series of consultations with a variety of professionals and members of public organizations. The process was an iterative one in which the UWCGPH consulted with individuals and, after each round of consultation, incorporated expert commentary into a working document.

### Professional consultant sampling

We sought consultation with professionals involved in asthma or genomics research and medical or public health practice. Experts were identified using a snowball sampling technique, beginning with Seattle area researchers, health care providers, and public health practitioners identified by the working group (2). These consultants were asked to recommend additional local and national experts; additional experts were sought until no new experts were identified. A total of 47 professionals who represented a broad range of organizations, disciplines, and interests participated in the asthma consultative process. This total does not include the 12 members of the Asthma Working Group.

### Professional consultant format

Consultations were conducted by two authors (Burke and Harrison) between January and September 2003 through telephone or in-person key informant interviews and group discussions. Consultations began with a description of the project, including both frameworks, either formally through a PowerPoint presentation for group consultations or informally for individual or small group discussions. During consultations, approximately 30 minutes to two hours in duration, participants were asked to comment on the frameworks and to address a set of open-ended questions developed by the working group. The questions focused on the potential implications genomics may have for asthma prevention, diagnosis, and management. Written notes were taken and group discussions were tape-recorded with the permission of participants. Some consultants requested additional background materials, including review papers, prior to consultative meetings.

### Community consultant sampling

Initial efforts to solicit expert opinion were directed toward professionals working in scientific, public health, or medical fields. However, as indicated in the framework, the perspective of community members is also vital to integrating genomics into public health and medical practice. The working group first identified local community organizations with an interest in asthma. The UWCGPH contacted each organization to assess its interest in participating in the asthma consultative process. A representative from the organization then approached the group's members and asked if they would be willing to participate in a group discussion about genomics and asthma. Because of time and funding constraints, only a subset of organizations identified by the working group were asked to participate in the process. Three community groups, including 18 community members, participated in the group consultations between September and October 2003. Approval for community consultations was obtained by the University of Washington's Human Subjects Division.

### Community consultants format

The UWCGPH worked with the community contacts to schedule the group meetings, to discuss the meeting environment, and to learn about the needs and interests of each group. A total of three community groups were consulted at three separate meetings held in the evenings. Foreign language translators were hired to assist with discussions for two meetings and to translate consent forms distributed to participants. Written notes taken by the UWCGPH were used to summarize discussions. Sessions were not tape-recorded.

The format for community consultations differed slightly from professional consultations. Community consultations consisted of group discussions led by a member of the UWCGPH and a representative from each organization. Each meeting began with a description of the project, followed by a general discussion about asthma and genomics. Participants were then read one to three hypothetical scenarios illustrating potential asthma-related uses of genetic information. The hypothetical scenarios were developed by the UWCGPH based on input provided by professional consultants about areas of asthma genomics that could potentially lead to health care applications. Topics represented by the scenarios were pharmacogenomics, newborn screening to identify susceptible individuals for possible prevention efforts, and policy development. Policy development centered on the use of genetic susceptibility information to assist in setting clean air standards. Scenarios were presented to the group and were followed by a discussion period where participants were encouraged to provide comment on the scenario, including identifying areas of concern. At the end of each meeting, participants were asked if they had any questions, were reminded that their input would be confidential, and were thanked for participation.

## Discussion

The UWCGPH developed the asthma consultative process to summarize asthma genomics research findings and examine the implications of these findings for public health research and practice. Results from this process, and from the literature review and working group discussions, have been compiled into a final report that is available from http://www.uwcgph.org/. Briefly, findings suggest that public health researchers and practitioners can ensure that genomics research supports public health goals by 1) facilitating the analysis and communication of research in asthma genomics, 2) promoting population-based research that investigates both genetic and environmental risk factors, and 3) conducting advocacy and outreach to promote access to genomics-based therapies and support community-based participatory research methods.

While the asthma consultative process described here was framed in the context of public health, it drew from experts involved in efforts to improve the population's health at several organizational levels and across several disciplines. The diversity of consultants was valuable to the process because it helped to 1) identify unanticipated issues that would not have been apparent from a single perspective or through a literature review alone, 2) identify unforeseen stakeholders, and 3) reflect the diversity of public health in our findings.

The asthma consultative process also allowed for a degree of flexibility. The scheduling of consultations with experts over several months, rather than at a single meeting, allowed for access to a wider range and greater number of experts and allowed us to tailor discussions to each consultant's area of expertise. Additionally, we were able to make efficient use of experts' time by consulting over the telephone, convening at national conferences, and scheduling group or individual appointments at locations and dates convenient for consultants. The iterative process also allowed us to return to issues during the course of the consultation as new insights emerged. Thus, the frameworks and report developed by the working group were continually revised after each consultation and over time led to a document that served as the basis for the final report.

Another advantage of the process was that it provided opportunities to gauge the level of awareness about public health genomics and to assist public health practitioners interested in learning about asthma genomics. Few consultants had considered genomics in the context of public health practice. In some instances, discussions enabled consultants to create new relationships and exchange ideas, providing them with opportunities to learn about research or public health practices and to gain insights into perspectives other than their own. Additionally, the process created opportunities for assisting public health practitioners to learn about asthma genomics. For example, the UWCGPH provided technical assistance on asthma genomics to three state health departments and developed Web pages highlighting information about asthma genomics as part of the CDC Public Health Perspective series.

The asthma consultative process also had some limitations. The process was a voluntary one in which experts were recommended by other consultants. Because of this, the participation of individuals representing each perspective could not be guaranteed. For example, a sixth perspective, that of the commercial developer, was identified during initial consultations and was added to the framework. Although individuals representing the commercial perspective were invited to provide consultations, none participated in the process. Additionally, resource and time constraints did not allow us to conduct as comprehensive a series of consultations with community groups as were conducted with professionals. Future efforts are needed to expand the dialogue about public health genomics with additional community organizations, asthmatic individuals and their families, and commercial developers.

In summary, the asthma consultative process offers an efficient approach for examining the potential implications that genomics will have for the field of public health. The method described in this paper not only enabled the UWCGPH to review the current state of knowledge in asthma genomics, but it allowed for opportunities to engage multiple stakeholders, create linkages among experts from different disciplines, and generate awareness about public health genomics.

The collaboration between professionals from multiple health-related disciplines will be fundamental to bridging the gap between the identification of genetic contributions to disease and the development of new genomics-based interventions to improve health outcomes. In an effort to promote this important collaboration, the UWCGPH carried out the asthma consultative process. Processes such as this one can serve to strengthen the capacity to integrate genomics into public health research and practice.

## Appendix. Consultation Guide Discussion Probes

1. How would you define genomics?
2. Does the framework provided by the translational pathway and the table provide a useful way of framing the discussion of the implications of genomics for asthma disease prevention?
  - Are there missing perspectives? What different groups/agencies do you see represented in each perspective?
  - Are there alternative ways to approach the problem?
  - What perspective(s) do you feel you represent?
3. From your perspective, is genomics currently a factor in asthma care? If yes, does it affect:
  - Universal prevention measures
  - Risk-based prevention measures
  - Diagnosis
  - Management
  - Public health efforts
  If no, why not and what would make it applicable? Are there barriers?
4. Is genomics likely to have an impact on asthma care in the future? If yes, will it affect:
  - Universal prevention measures
  - Risk-based prevention measures
  - Diagnosis
  - Management
  - Public health efforts
  If no, why not and what would make it applicable? Are there barriers?
5. What research is most important in the area of asthma genomics? Why is this research most important?
  - What are the barriers to accomplishing this research?
  - What could be done to encourage this research?
  - How could this research contribute to improved asthma outcomes?
6. Are there potential harms related to asthma genomics?
  - If yes:
    - What are they?
    - Are there potential mechanisms/solutions to prevent or control these harms?
  - If no, why not?
7. Does your own work involve genomics?
  - If yes, how?
  - If no, do you expect it to do so in the future?
8. Do state agencies and the CDC have a role in the application of genomic information or research to asthma disease prevention?
  - If yes, how?
  - If no, why and do you expect it to do so in the future?
